# Challenges in Identifying the Retracted Status of an Article

**DOI:** 10.1001/jamanetworkopen.2021.15648

**Published:** 2021-06-29

**Authors:** Elizabeth M. Suelzer, Jennifer Deal, Karen Hanus, Barbara E. Ruggeri, Elizabeth Witkowski

**Affiliations:** 1 Medical College of Wisconsin Libraries, Medical College of Wisconsin, Milwaukee; 2 Advocate Aurora Library, Advocate Aurora Health, Milwaukee, Wisconsin; 3 Todd Wehr Library, Carroll University, Waukesha, Wisconsin

## Abstract

This cross-sectional study examines publisher websites and bibliographic databases to check their accordance with industry standards for documenting retracted publications.

## Introduction

Inadvertent or unacknowledged citations to retracted literature are a persistent problem in scholarly publishing.^1,2^ Inconsistencies in how retraction information is displayed on publisher websites and bibliographic databases hinder determining whether a paper has been retracted, thus contributing to the cause of this problem. This cross-sectional study examines publisher websites and bibliographic databases to check their accordance with industry standards for documenting retracted publications, highlighting challenges readers face in identifying the retracted status of a publication.

## Methods

A search was performed in PubMed on October 20, 2019, to identify English-language articles published between 2009 and 2019 that were indexed with a publication type “Retracted Publication.” Three articles were selected from the 50 journals that had the most retracted publications in the PubMed search, giving us 150 articles for analysis.

Articles were examined on publisher websites to document whether the websites followed specific recommendations from the International Committee of Medical Journal Editors (ICMJE) on documenting retractions,^3^ and we looked for consistency within the websites on how this information was displayed. In accordance with guidelines from Committee on Publishing Ethics (COPE) on retraction,^4^ the identification of retractions in bibliographic databases were examined. The same articles were reviewed in 6 bibliographic databases that index biomedical literature (PubMed, Ovid MEDLINE, EBSCO CINAHL, ProQuest PsycINFO, Scopus, and Web of Science) to document how the databases displayed retraction information using PubMed’s criteria for updating retracted articles.^5^ Detailed methods describing the literature search, review, and data abstraction can be found in the eAppendix in the Supplement.

This study followed the Strengthening the Reporting of Observational Studies in Epidemiology (STROBE) reporting guideline,^6^ and a descriptive analysis of the data was performed in Excel (Microsoft Corp). The Medical College of Wisconsin institutional review board office reviewed this project and determined that this study did not qualify as human participants research and was therefore not subject to institutional review board review.

## Results

Owing to the varying scope of the databases in our study, not all articles were indexed across all databases, and coverage ranged from 100% (150 articles) in PubMed and Ovid MEDLINE to 4% (6 articles) in ProQuest PsycINFO. Performance varied among the 6 bibliographic databases. PubMed and Ovid MEDLINE showed the best performance in adhering to their procedures for updating retracted publications with 87% (650 of 750) compliance with all 5 criteria, whereas ProQuest PsycINFO’s performance was 23% (7 of 30) and EBSCO CINAHL was 7% (8 of 115) (Table 1).

**Table 1.** Database Adherence With Following PubMed’s Retraction Procedure^a^

Database	Total No. of Articles	Articles, No (%)					Overall score, No./total No. (%)
		Adds retraction label	Includes or links to retraction notice from original article	Includes or links to original article from retraction notice	Document or publication type changed	Indexing: title and authors consistent in notice and original article	
PubMed	150	150 (100)	147 (98.0)	147 (98.0)	150 (100)	56 (37.3)	650/750 (86.7)
EBSCO CINAHL	23	0	0	0	4 (17.4)	4 (17.4)	8/115 (7.0)
Ovid MEDLINE	150	150 (100)	147 (98.0)	147 (98.0)	150 (100)	56 (37.3)	650/750 (86.7)
ProQuest PsycINFO	6	3 (50.0)	4 (33.3)	4 (33.3)	0	0	7/30 (23.3)
Scopus	146	73 (50.0)	73 (50.0)	67 (45.9)	45 (30.8)	74 (50.7)	332/730 (45.5)
Web of Science	138	82 (59.4)	58 (42.0)	103 (74.6)	0	104 (75.4)	430/690 (62.3)

^a^ The frequency of databases incorporating the steps of PubMed’s retraction procedure for updating retracted articles records. The numbers indicate how many article records incorporated the criteria used by PubMed, and the overall score looks at actual adherence divided by potential for adherence of articles in the different databases.

PubMed, the only free database in our analysis, had the highest performance in documenting retracted publications. ProQuest PsycINFO and EBSCO CINAHL often failed to use labels to indicate an article’s retracted status, such as updating the publication type to retracted or linking to the notice of retraction. Lack of consistency between bibliographic databases results in some users being better informed than others, depending on which database they can access.

Overall, 112 of 150 articles (75%; 95% CI, 67%-81%) on the publisher websites followed at least 6 of the ICMJE’s 7 recommendations for reporting retractions and demonstrated variability in where publishers displayed the retraction information (Table 2). When we compared the retraction labels within the publisher websites using 3 examples from each of the 50 journals, retraction information was consistently displayed in all 3 of the article abstracts 78% (39 of 50) of the time and 64% (31 of 50) of the time in PDFs. Examples of inconsistencies included: links to the notice of retraction not present on all examples, location of the retraction notice differed, different colors were used to show retraction labels, and some examples did not show retraction information.

**Table 2.** Labeling of Retracted Articles on Publisher Websites

Criteria^a^	Articles, No./total No. (%)
1. Original article title is used in the notice of retraction title	130/150 (86.7)
2. Retracted article’s abstract is labeled	141/147 (95.9)
3. Retracted article’s abstract links to notice	127/145 (87.6)
4. Notice of retraction appears in TOC	142/150 (94.7)
5. Notice of retraction links to retracted article	109/148 (73.6)
6. Retracted article’s HTML full text is labeled	110/113 (97.3)
7. Retracted article’s PDF is labeled	132/150 (88.0)
Meets all the criteria	70/150 (46.7)
No. of ICMJE criteria met	
7	70/150 (46.7)
6	42/150 (28)
5	13/150 (8.7)
4	13/150 (8.7)
3	8/150 (5.3)
2	4/150 (2.7)
Location of article’s retracted status on publisher’s website^b^	
“Retraction” added to article title	44/150 (29.3)
Label or notice posted above article title	33/150 (22.0)
Label or notice between the article title and citation	3/150 (2.0)
Under citation, above the abstract	89/150 (59.3)
Under the abstract	9/150 (6.0)
Right-side navigation	3/150 (2.0)
Bottom of the webpage	4/150 (2.7)
Uses more than 1 label	44/150 (29.3)
No label was used	9/150 (6.0)

Abbreviations: ICMJE, the International Committee of Medical Journal Editors; TOC, table of contents.

^a^ Websites that did not include an abstract or an HTML or PDF version of the article were not included in the total calculations.

^b^ Location of the retraction notice is related to the ICMJE recommendation 2 (ie, retracted article’s abstract is labeled). For a schematic representation of the location of the retraction, see eFigure in the Supplement.

## Discussion

In this study, journal websites and bibliographic databases did not consistently display the retracted status of articles. Guidance on retracting articles is given by the Committee on Publishing Ethics and the ICMJE,^3,4^ but their recommendations are open to interpretation and not always followed. The ICMJE recommends that retractions should be “prominently labelled,”^3^ and we propose that an explicit recommendation to add a prefix of “Retracted:” to the title of a retracted publication would fulfill this aim. This change would provide a consistent visual signal to the reader and would change the metadata that can be ingested into citation managers.

Our study is limited by the number of retracted articles that were reviewed (150 of the entire data set of 7059 articles), and Embase was not included in this study because the authors did not have access to it.

We hope that by making the retraction information more clearly discoverable and standardized, the number of unintentional and unacknowledged citations of retracted literature will be reduced.

## Notes

Supplement. **eAppendix.** Methods eReferences. **eFigure.** Schematic Representing Placement of Retraction Labels on Publisher Websites
