# Errors in Table 1

**DOI:** 10.1001/jamanetworkopen.2022.3513

**Published:** 2022-02-28

In the Research Letter titled “Challenges in Identifying the Retracted Status of an Article,”^1^ published June 29, 2021, there were errors in Table 1. For the ProQuest PsychINFO database, there were 2 articles (33.3%) that included or linked to a retraction notice from an original article and 2 articles (33.3%) that included or linked to an original article from a retraction notice. For the Web of Science database, there were 83 articles (60.1%) in which the document or publication type changed. The overall scores were correctly stated, and these 3 errors do not affect the conclusions. This article has been corrected.^1^
