# Supplementary material for: Challenges in Identifying the Retracted Status of an Article
Source: JAMA Netw Open. 2021 Jun 29;4(6):e2115648. doi: 10.1001/jamanetworkopen.2021.15648 (PMC8243230; doi:10.1001/jamanetworkopen.2021.15648)
Supplement: Supplement. — eAppendix. Methods eReferences. eFigure. Schematic Representing Placement of Retraction Labels on Publisher Websites [file jamanetwopen-e2115648-s001.pdf]

Supplemental Online Content

Suelzer EM, Deal J, Hanus K, Ruggeri BE, Witkowski E. Challenges in identifying the retracted status of an article. *JAMA Netw Open*. 2021;4(7):e2115648.
doi:10.1001/jamanetworkopen.2021.15648

eAppendix. Methods

eReferences.

eFigure. Schematic Representing Placement of Retraction Labels on Publisher Websites

This supplemental material has been provided by the authors to give readers additional information about their work.

© 2021 Suelzer EM et al. *JAMA Network Open.*

eMethods

Article Selection

To determine which articles to review, a search was conducted in PubMed for items indexed with a publication type "Retraction of Publication" on Oct. 20, 2019, and results were limited to articles published in English since 2009. All 7059 citations were exported into Excel where we identified the 50 journals with the most retracted articles. European Review for Medical and Pharmacological Sciences showed up in our list, but this journal was excluded because reviewers were not able to access the PDFs of articles for analysis.

Reviewers (JD, KH, BR, ES, EW) selected three articles from each of the 50 journals to analyze. A point was made to choose three retracted articles from each journal that were published in different years, looking specifically for the earliest and latest date published, and an article from a year in-between. In some cases, retractions occurred in the same year, so we were unable to select citations across the time span.

Reviewers (JD, KH, BR, ES, EW) independently reviewed information from the publisher websites in March 2020 and bibliographic databases in June 2020 and populated Excel spreadsheets for data analysis. Data extraction were checked for consistency by two reviewers. (KH, BR).

Data Analysis

For the publisher website analysis, we collected citation information such as journal name, DOI, and publisher; location and visual description of the retraction notice in the abstract and HTML and PDF version of the article that was posted on the publisher's website; and whether the seven recommendations from the ICMJE were being followed.

The seven recommendations are: retractions should "[1] appear on an electronic or numbered print page that is included in an electronic or a print Table of Contents to ensure proper indexing, and [2] include in their heading the title of the original article. [3,4] Online, the retraction and original article should be linked in both directions and [5,6,7] the retracted article should be clearly labelled as retracted in all its forms (Abstract, full text, PDF)."[1]

For the bibliographic database analysis, we collected citation information such as journal name and article DOI; we indicated if the article was indexed in the database, and if so, the following five items were documented:

1. Adds retraction label to the retracted article
2. Publication type changed to "retracted"
3. Retracted article links to retraction notice
4. Retraction notice links to retracted article
5. Title & authors consistent in both retraction notice and retracted article

These items come from PubMed's procedure[2] for documenting retracted publications and we included the recommendations from COPE[3] and ICMJE to check for consistency in the authors and title in both the retraction notice and retracted article. An overall score was given for each database to document how many times PubMed's retraction criteria were followed in the article records divided by the potential number of times that the criteria could have been followed.

© 2021 Suelzer EM et al. *JAMA Network Open.*

References

1. International Committee of Medical Journal Editors. Recommendations for the Conduct, Reporting, Editing, and Publication of Scholarly Work in Medical Journals. Published online December 2019. Accessed May 14, 2020. http://www.icmje.org/recommendations/

2. National Library of Medicine. Errata, retractions, and other linked citations in PubMed. Accessed June 16, 2020. https://www.nlm.nih.gov/bsd/policy/errata.html

3. COPE Council. *COPE Retraction Guidelines*. Committee on Publication Ethics; 2019. doi:10.24318/cope.2019.1.4

© 2021 Suelzer EM et al. *JAMA Network Open.*

eFigure 1: Schematic Representing Placement of Retraction Labels on Publisher Websites

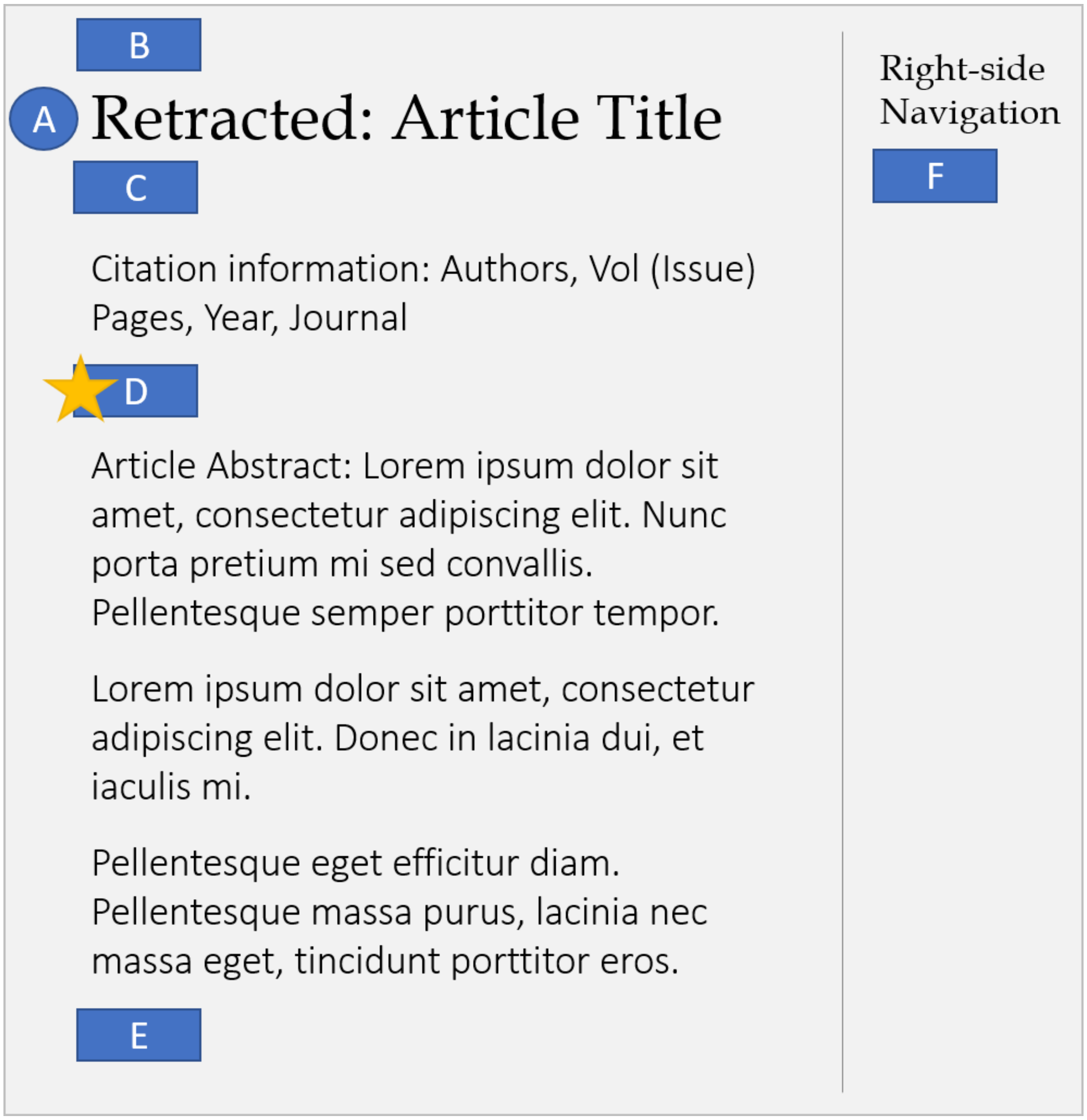

Retraction label locations (N=150): A: title changed, 44; B: above article, 33; C: between the article and citation, 3; D: under the citation and above the abstract, 89; E: under the abstract, 9; F: right-side navigation, 3; bottom of the webpage, 4; used more than one label, 44; no indication of retraction, 9.
